# The role of social support and social contact in cognitive aging: A study of sex/gender and sexuality in the Health and Retirement Study

**DOI:** 10.1002/alz.089272

**Published:** 2025-01-09

**Authors:** Douglas William Hanes, Arne Stinchcombe, Sean A.P. Clouston

**Affiliations:** ^1^ Renaissance School of Medicine at Stony Brook University, Stony Brook, NY USA; ^2^ University of Ottawa, Ottawa, ON Canada; ^3^ Bruyère Research Institute, Ottawa, ON Canada; ^4^ Stony Brook University, Stony Brook, NY USA

## Abstract

**Background:**

Sexual‐minority (e.g., lesbian, gay, bisexual [SM]) people may be at an increased risk of Alzheimer’s disease or a related dementia (ADRD) from stress related to experiences of minoritization. Strong levels of social contact and social support decreases dementia risk; these effects may differ by sex/gender and sexuality. The purpose of this presentation is to elucidate the relationship between cognitive function, social support, and social networks with a focus on sexuality and sex/gender.

**Method:**

Data analyzed were from waves 8‐14 (2006‐2018) of the Health and Retirement Study, a longitudinal survey of U.S. residents aged ≥51 years (N = 18,183). Participants who reported their sexual orientation as lesbian, gay, or bisexual, or those in same‐sex relationships for at least one wave were classified as SMs. A gender/sexuality variable was created with four levels: SM‐women (n = 101), heterosexual women (n = 9,942), SM‐men (n = 106), and heterosexual men (n = 8,034). Social contact and support were reported for network size, frequency of contact, and quality of interactions across four relationships: spouses, children, family, and friends. Cognitive function was measured on a 27‐point scale. The primary analysis consisted of a series of multiple linear regression models, treating cognition as the outcome, and adjusting for age, wealth, education, and race.

**Result:**

Descriptive analysis showed that SM‐men and SM‐women tended to be younger and join the study later than heterosexual men and women, and at baseline, heterosexual men had lower cognitive scores compared to the other three groups (F = 82.44, p<0.001). Moderation analysis showed the relationship between positive spousal support and cognition was inconsistent across gender/sexuality groups (χ^2^ = 11.58, p = 0.009); SM‐women did not benefit from positive spousal support as much as other gender/sexuality groups. Greater frequency of social contact was associated with higher cognitive function, except among SM‐women where greater social contact was associated with lower cognitive function (χ^2^ = 9.65, p = 0.023). Support from other social groups did not have differential effects across gender/sexuality groups.

**Conclusion:**

Findings highlighted variable and gendered effects of social support and social contact on cognitive functioning in aging SM people. Examining differential effects for SM/gender groups is essential to inclusive ADRD research.

